# The Hospital. Nursing Section

**Published:** 1905-04-22

**Authors:** 


### The Hospital.

### Nursing Section.

Contributions for this Section of "The Hospital" should be addressed to the Editor, "The Hospital" Nursing Section, 28 & 29 Southampton Street, Strand, London, W.C.

No. 969.—Vol. XXXVIII. SATURDAY, APRIL 22, 1905.

### Notes on News from the Nursing World.

### NURSES AND THEIR UNIFORM.

The proposal of "A Trained Nurse" in our issue of April 8th, in which she suggested as a remedy for the wearing of nurses' uniform by untrained women, and the attack on the appearance of trained nurses which it provoked last week, have evidently excited a good deal of interest. Among our readers, we are afraid that neither the scheme of "A Trained Nurse" who urges that each certificated nurse should wear a medal with the initials "C. N." on the right shoulder, nor that of another correspondent this week who thinks that the difficulty in distinguishing partly and fully-trained nurses in all branches might be got over by the use of a sort of graduated cuff denoting the period of training, is practicable or would at all improve the situation. In passing, we must express surprise at the statement of the latter correspondent who, writing with the authority of an official of an important organisation, affirms that doctors in her district frequently employ cottage nurses for operations because there are no others at hand. We admit that in a case of life and death it is better to employ a cottage-trained nurse than none, but this should not be a normal condition of affairs as it appears to be in our correspondent's district. With regard to the question whether trained nurses are untidy in their appearance out of doors, we agree with "An American-Trained Nurse," that this should be discussed in a charitable spirit by those who differ in opinion. There is no doubt that a proportion of nurses, whose training is unimpeachable, do not dress as trimly as they might when they are out of doors; and we think that nurses generally cannot be too careful to protect themselves from the charge of being untidy and slovenly. If unqualified persons dishonour the uniform by appropriating it without right, those who are entitled to the uniform should scrupulously honour it by wearing it becomingly. But the assertion that only the nurses of a particular London hospital are immaculate in their uniform, is obviously inaccurate. It is only fair to other training schools to say that tidiness in dress and appearance are certainly not monopolised by the graduates or probationers of a single institution, and the author of such an assertion does much to put herself out of court as a critic.

### THE UNTRAINED MATRON AT WORK.

The proceedings at an inquest at St. Albans Workhouse reported in a local paper last Saturday merit remark. The deceased was an elderly man who on his arrival at the workhouse was taken by order of the matron to the infirmary. He had been previously admitted on several occasions, and was supposed to be suffering from alcoholism. The head nurse took his temperature, administered brandy, and made him as comfortable as she could; and the night nurse who subsequently attended him gave him the milk and brandy as she thought necessary. He slept at intervals but shortly after midnight he became worse and died. The medical man who made a post-mortem examination of the body attributed death to syncope, the result of long-standing disease, and the jury returned a verdict accordingly. But the Coroner in summing up the evidence not only strongly criticised the action and inaction of the two nurses at St. Albans Infirmary, but attacked the entire nursing profession. We think that he would have done better if he had concentrated his attention on the points which show the necessity of reform in the nursing arrangements. Of course a medical man should have been informed of the admission of the old man, and equally, of course, brandy should not have been administered to a person supposed to have been suffering from alcoholism. But a trained matron would at once have telephoned for the doctor—the workhouse infirmary is connected with the doctor's private house by telephone—and either she would have perceived that the patient was not suffering from alcoholism, or else would not have allowed brandy to be given to him. We do not blame the matron at St. Albans, who doubtless did what she thought was best in the circumstances; but the incident demonstrates anew the importance of the official in authority over the nurses of an institution being herself a fully-trained nurse, if only in order that, in the event of disaster, there may be no difficulty in placing the responsibility for mistakes upon the right shoulders.

### DISTRICT NURSING SUBSCRIPTIONS.

A paragraph in the report which was read at the annual meeting of the Ryde District Nursing Association touches on a subject of general importance. It was announced by the treasurer at the monthly meeting in December that the available funds were insufficient to meet the expenses to the end of the year, and it was therefore most reluctantly decided to draw on the deposit for that purpose. Meanwhile, however, a vigorous effort was made to get in all outstanding subscriptions for 1904, and this was so successful that in consequence, with the assistance of some donations, the committee were able at the end of the year to replace the deposit. "It would be an immense help," the report added, "if when possible subscribers would be kind enough to send their subscriptions in early in the year." Many other committees, we are sure, would be glad if their subscribers would be more prompt in forwarding their contributions. "He gives twice who gives quickly," and it would save the managers of district nursing associations much trouble and perhaps considerable anxiety if subscriptions were all paid during the first month in the year. But those who do not pay by that time should be politely reminded that their subscriptions are overdue.

### LACK OF NIGHT NURSES IN YORKSHIRE.

In his Nursing Return for 1904, Mr. P. H. Bagenal, Local Government Board Inspector for the East and West Ridings of Yorkshire, states that the nurses on day duty at the end of the year numbered 316, and on night duty 117, an equivalent of 1,195 patients per nurse. The pauper attendants numbered 124. With regard to the proportion of nurses to patients, the inspector mentions that the position was practically the same as in the previous year. The pauper attendants, however, for 1903 numbered only 84, which unfortunately indicates retrogression instead of progress. A more serious matter, as Mr. Bagenal himself points out, is that there are still in the East and West Ridings no fewer than 16 workhouses in which it has not been found possible to provide a night nurse. We regret that there should be so many guardians of the poor in Yorkshire who do not yet realise the fact that it is essential in the interests of sick paupers that there should be at least one nurse employed on night duty in every institution under their control.

### THE NURSE AND THE PUMP.

At a meeting of the Pershore Guardians the chairman stated that the assistant nurse at the sanatorium objected to pump water. He went on to say that "it was simply abominable to have to keep a man to help two nurses"; and he moved that if the assistant nurse did not pump the water required she must have notice to quit. It appears that there are only five patients at the sanatorium, and so long as this is the case it cannot be said that the two nurses are overworked. It is not usual to expect a nurse to pump water, but we see no reason why she should not do it as an act of grace in a small institution where a spirit of co-operation is essential.

### A NURSING SUNDAY AT GRIMSBY.

In presiding at the annual meeting of the Grimsby and District Nursing Institution, Lord Yarborough said it was very gratifying to learn from the report that there had been a great increase in its receipts. He noted with special satisfaction the fact that the contributions from grateful patients amounted to nearly £30. He was also glad to see that Grimsby during the year had not suffered greatly from typhoid like the capital city of the county. The Rev. J. F. Quirk, in seconding the adoption of the report, suggested that one Sunday in the year should be set apart for collections at the churches to be devoted to the institution in a similar way to those of Hospital Sunday. This is an admirable idea, if it can be carried out. As to the work of the Grimsby nurses, they attended 420 cases during the year, and paid 12,223 visits. The Vicar of Grimsby subsequently called attention to the case of persons who need a nurse but cannot pay the fees of private nurses. He inquired if something could not be done by the institution to meet the want. We realise that this want is very often felt, but we are glad that no formal proposal was put forward to combine private with district nursing under the auspices of the Grimsby Institution.

### AN OVER-WORKED NURSE.

The nurse at Torrington Workhouse Infirmary has tendered her resignation on the ground that the duties are more than she can stand. She attended the meeting of the Guardians and informed them that she was not only on duty from 6 A.M. to 9 P.M. daily, but was liable to be called upon for duty at any hour. She also complained that she did not get sufficient time to eat her breakfast. The guardians decided, in accepting her resignation, to give her a testimonial and to issue advertisements for another nurse. It is to be hoped that if they get one, they will see that she is treated more considerately.

### MIDWIFERY AND CLEANLINESS.

In an address delivered last week before the Incorporated Society of Medical Officers of Health, Dr. G. H. Fosbrooke dwelt on the necessity of cleanliness on the part of midwives. Although it was one of the most important qualifications for the work, he said that his experience in Worcestershire was that 8 per cent. of the midwives were very dirty in person or clothes, that 10 per cent. lived in filthy homes, and that 16 per cent. wore unwashable clothes. One Worcestershire midwife he knew stored her appliances in a luncheon-basket, and another in a box containing a syringe used to treat an ulcer. No doubt many instances of this kind might be given, but it may fairly be hoped that even if the midwives eligible under the new Act are disposed to ignore indispensable conditions of cleanliness, the inspectors will take care that they are observed.

### CHANGES IN THE MILITARY NURSING SERVICE.

We are officially informed that Miss A. Garriock, R.R.C., has been appointed matron of the Royal Herbert Hospital, Woolwich, on her return from Indian troopship duty. Miss E. C. Humphreys, sister, has been posted to the Royal Herbert Hospital, Woolwich, on her return from Indian troopship duty; and Miss M. R. Makepeace, sister, to the Royal Victoria Hospital, Netley, on her return from South Africa. Miss M. Brown and Miss E. C. Ellis have been appointed staff nurses in Queen Alexandra's Imperial Military Nursing Service. Miss S. O. Beamish, has been posted to Station Hospital, Shorncliffe, for temporary duty; Miss E. M. Goard and Miss E. J. Minns, to Connaught Hospital, Aldershot, as staff nurses. Miss E. Barber and Miss B. F. Perkins, staff nurses, have been transferred to Malta from the Connaught Hospital, Aldershot, and Miss E. M. Lang, staff nurse, to the Royal Victoria Hospital, Netley, from Lincoln. The appointment of Miss M. Smith as sister has been confirmed; and also the appointment of Misses F. A. Dawson, A. M. M. Denny, F. G. P. de S. Zrinyi, E. M. Fairchild, O. M. Griffin, E. M. Keays, E. M. Lyde,

E. L. McAllister, A. M. MacCormac, E. M. Perkins, G. M. Smith, and M. E. Wilkin as staff nurses.

### THE SITUATION AT LINCOLN.

We understand that the nursing of typhoid fever patients in their own homes at Lincoln is now greatly lessened, owing to the decrease of cases, and that the staff of extra district nurses has consequently been much reduced in number. As an example of the improvement we may state that, in one group of three parishes seven weeks ago, 163 cases were being daily attended in their own homes by district nurses; at the present time, in the same group of parishes, the number of patients being nursed is 30. As to the nurses in the hospitals, the number at present remains the same. The staff in the large Drill Hall Hospital has been slightly diminished, as it was found advisable to admit fewer patients, but in the smaller hospitals more beds have been added, and therefore more nurses. The work of supplying food and other comforts at the various depôts is also very materially lessened, and although Miss Bromhead and her colleagues have plenty of work to do they are glad to feel that the strain imposed upon them is steadily relaxing.

### NEW HOME FOR FULHAM POOR-LAW NURSES.

A new Home for the nursing staff of the Fulham Union Infirmary will be opened by Princess Christian early in July. It occupies a corner site in Fulham Palace Road, immediately opposite the Infirmary buildings. Consisting of three floors and a basement, it is built in the shape of the letter L, the space between the wings being intended for gardens and tennis-lawn. There are 63 separate bedrooms, including a spare room, home sisters', and visitors' room. All are very light, and those on the side facing the grounds have a south aspect. The building is heated with hot-water coils, and, in addition, each bedroom is provided with an open tiled fireplace. Entirely new furniture will be provided, and that at present in use will be relegated to the ward maids, a staff of whom is being engaged. Picture rails have been placed in every room. The floors will be covered with linoleum. The top floor will be devoted to the night nurses. The many-windowed, spacious sitting-room for sisters and nurses is separated by folding doors; the floor will be stained, and seats are being fixed to the walls. A small room opening out of the sisters' room is called "The Nook." The large dining-room adjoining is served by means of a lift from the white and green tiled kitchens on the basement, and an entirely separate domestic staff is being engaged. A small room is being fitted up as a library. Facing the grounds is a *loggia*, where seats, plants, etc., are to be provided. Ample linen, airing, and bath-rooms—the latter white tiled—have been arranged for, and there is a large bicycle-room, which is reached by a track from the road. Electric fittings are being installed, and a subway connecting the Home with the Infirmary will complete the arrangements.

### AN URGENT NEED AT OBER AMMERGAU.

The writer of the article on the Ober Ammergau Cottage Hospital, which appears in another column, assures us that the sister in charge must often be overwhelmed with work; and worse than this, our correspondent says that she has known precious lives lost for want of a qualified nurse. There could not be a more appropriate season of the year than this for those who have derived pleasure and profit from visiting the beautiful and world-renowned spot where the Passion Play is performed, to mark their appreciation of the privilege they have enjoyed by contributing to the fund which is being raised for the endowment of a second nurse.

### THE MATRONSHIP OF MALVERN RURAL HOSPITAL.

At the annual meeting of the Malvern Rural Hospital the appointment of the new matron was referred to. The committee stated in their report that out of 138 applications for the post of matron, Miss Harrison had been selected. Her testimonials, it was stated, were of a very high order, and the committee expressed their conviction that under her management the hospital would prove of great benefit to the sick poor of Malvern.

### IRISH NURSES' ASSOCIATION.

An interesting and instructive lecture on fevers was delivered last week by Dr. Moorhead, one of the visiting physicians of the Royal City of Dublin Hospital, to the members of the Irish Nurses' Association. At the close of the lecture a vote of thanks was proposed by Sister Burke, seconded by Sister O'Clery, and conveyed to Dr. Moorhead by Mrs. Kildare Tracey, lady superintendent of Dublin Nursing Institute, who presided.

### NURSES AS HOSTESSES.

During the visit of the Royal Sanitary Institute to Ham Green Hospital, Bristol, the matron, Miss Garden, and her staff of nurses made delightful hostesses, and looked well after the comfort and refreshment of the visitors.

### THE AMENDMENT OF THE MIDWIVES ACT.

At the last meeting of the Cambridge Guardians, Dr. Dalton intimated that he desired to withdraw a resolution of which he had given notice to the effect, "That the district medical officer be informed that when summoned by a certificated midwife to attend an emergency, and there is not time to apply to the relieving officer, they attend the case as if they had been furnished with an order of the relieving officer, in such case the relief to be given on loan unless the Board decide otherwise." Dr. Dalton explained that the reason he did not propose to proceed with the resolution was that the Central Midwives Board hope to get a Bill dealing with the difficulty through Parliament shortly.

### INCIDENTS IN A NURSE'S LIFE.

Every nurse of standing must in the course of her experience have met with many interesting incidents which are well worth recording. We shall always be glad to receive accounts of any such incidents as our readers may send for publication. They should be written on one side of the paper only and not exceed 500 words in length. All such copy, if accepted, will be paid for.

### The Nursing Outlook.

"From magnanimity, all fear above;
From nobler recompense, above applause,
Which owes to man's short outlook all its charm."

### NURSE TRAINING IN LONDON.

There can be no doubt in the general meaning of the words that the training given at the larger hospitals in London is on the whole satisfactory, so far, at any rate, as the present system permits. The period of training differs in the sense, that at some of the hospitals, like the London and Guy's, preliminary training is given before a probationer is accepted for admission to the nurse-training school. In the majority of hospitals with medical schools the period of training extends over three years, but in some of them the certificate is not granted until an additional year's service has been rendered to the hospital, that is, at the end of the fourth year. At the London there is a few weeks' preliminary training, then two years as a probationer when a first certificate is granted, and then two years' further service with the institution, and a final certificate at the end of the fourth year. At St. Thomas's a nurse acts as probationer for one year, and then serves as a staff nurse for three subsequent years. Three years' probation is the rule, and if the certificate be withheld until the end of the fourth year, that is a matter of contract between the probationer and the hospital authorities. The training in the wards is carried on under the supervision of the sisters, and is supplemented by courses of lectures which are given by the matron or her assistants and by members of the honorary medical staff. The first year is usually devoted mainly to preliminary instruction in the duties of a nurse in a ward, and the last two years are given up to practical courses in children's and special departments, and in some cases to private nursing through the institution attached to the hospital. The examinations are usually both *viva voce* and written; in some cases they are held each year, in some at the termination of each course of lectures, and in some less frequently, in accordance with the plan of instruction adopted by the authorities of each hospital. It is fair to say, again speaking generally, that the methods pursued at the chief London hospitals are satisfactory, and that any nurse who is fortunate enough to obtain one of their certificates should be entitled to consider herself fully trained and efficient.

Action still is needed to bring about certain changes which would add to the efficiency of the training, and afford reasonable satisfaction to the nurses and the public who employ them. All the nurse-training schools should have an accepted uniform curriculum of training, uniform certificates, and should publish evidence, that each hospital provides, that every probationer does, in fact, receive methodical instruction in all branches of her work. We urged last week the importance of affiliating the nurse-training schools and of securing co-operation between them and all other institutions where nurses may at present receive some portion of their training. The anomaly whereby special and fever hospitals grant certificates, after requiring two or three years' service from their nurses, though they cannot in fact afford nurses methodical instruction in all branches of their work, should be abolished. Steps should be taken to make it impossible for any hospital to allow a school to use its nurses for the efficient working of the hospital, without regard to the training of each nurse. It should be impossible, too, that some probationers should remain for months in one ward and never see special work—*i.e.* children's, ophthalmic, gynæcological cases—and that they should be employed in the linen-room and on the domestic side of the hospital for an undue time. It is further unjustifiable to send out as private nurses any probationer before she has completed her hospital training in its entirety. Perhaps it is natural, but it is very undesirable, that there should be a tendency, in certain hospitals, to retain the smart and methodical nurses, and to let the least successful and consequently least completely trained go out from the schools to work for the general public.

All these points demand attention, and it is to the interest of every nurse-training school to show, that so far as their system of training is concerned, it effectually prevents the continuance of any of the objectionable features which we have just enumerated. Those authorities, which boldly take the bull by the horns, and declare, that they recognise the responsibility of giving each probationer who is accepted for training, a full and complete nursing education, will necessarily take the lead, and exercise the greatest influence over public and nursing opinion. The demand for nurses is so great, and the increase in the numbers trained each year so expanding in quantity, that it is essential, that all who undertake to train nurses should prove clearly, in the prospectuses of their schools, that they have put their house in order, and do in fact offer the fullest opportunities for methodical training in every branch of nursing. The time has gone by, when a hospital can decline any responsibility to give its probationers a full and complete nursing education, and where, for instance, no instruction is given in typhoid cases, for the reason, that no such patients are admitted to treatment. The latter is only one instance of the absence of responsibility which some authorities have exhibited in the past, but it illustrates the direction in which reform is called for, and we have no doubt will be introduced, either by affiliation, or through the spontaneous action of the authorities immediately concerned.

### The Nursing of Infectious Diseases.

By F. J. Woollacott, M.A., M.D., B.S.Oxon, D.P.H., Senior Assistant Medical Officer, Park Hospital, Metropolitan Asylums Board, Hither Green.

### LECTURE XIX.—SOME SYMPTOMS AND COMPLICATIONS OF ENTERIC FEVER.

We have already seen that an important feature of enteric fever is the presence of ulceration in the intestines, and, as might be expected, the abdominal symptoms are usually prominent throughout. As a rule there is more or less *diarrhœa*, the stools being liquid and of a light yellow colour. In most cases this will need no special treatment, but if excessive, *i.e.* if there are more than four or five stools in the twenty-four hours, or if it is exhausting to the patient, means must be taken to check it. Beef tea and all animal broths are removed from the diet, and lime water may be added to the milk. Various drugs are occasionally given by the mouth, but the most efficient remedy is the administration of a starch and opium enema. It should be quite cold and given very gently by means of a catheter and funnel or glass syringe. In many cases there is no diarrhœa but, on the other hand, *constipation*. This also needs attention. Aperients by the mouth are rarely advisable, owing to the danger of setting up energetic movements of the intestines and so acting injuriously on the ulceration, and it is consequently necessary to rely on enemata. These usually consist of one to two pints of warm water in which soap has been rubbed up, and they should be given by means of a Higginson's syringe, the nozzle of which has been well oiled. Occasionally an enema of glycerine or oil may be ordered. Plain cold water enemata, once or twice a day, are often serviceable, for they not only relieve the constipation, but to some extent tend to reduce the temperature. It is usual to secure an action of the bowels at least every other day. The stools should always be examined. They may contain undigested food, such as milk curds, or perhaps blood or pieces of slough from the ulcers in the intestine. If any of these or anything else unusual is noticed the stool should be saved for the inspection of the medical attendant. The condition of the abdomen is important. As a rule it is somewhat full, and very frequently there is considerable flatulent *distension.* This may be the case whether there be diarrhœa or constipation, and gives rise to discomfort and perhaps considerable distress by pressing upwards on the heart and lungs and so interfering with their action. The distension may often be relieved by the administration of a turpentine enema and the application to the abdomen of hot fomentations, sprinkled with a few drops of turpentine, or of fomentations prepared by wringing flannel out of hot water, to which a drachm of turpentine has been added. If other measures fail the introduction of a long tube into the rectum may enable the flatus to escape, and so afford relief. In some cases *vomiting* occurs and may prove very troublesome. The food should then be given in small quantities and frequently; and it may be necessary to peptonise the milk or to provide some substitute such as raw meat juice.

Owing to the presence of ulcers in the intestine two complications may arise, both of a very serious character. An ulcer may eat its way into a blood-vessel and cause *hæmorrhage*, or may extend deeper still and penetrate right through the intestinal wall and open into the peritoneal cavity. The latter complication is called *perforation.*

*Hæmorrhage.*—This is comparatively common and usually occurs during the height of the disease, *i.e.* about the third week. The amount of blood lost may be very small and only detected by careful examination of the stools. Under these circumstances there are no special symptoms and no treatment is required. On the other hand the amount may be so great as to endanger the patient's life. The stools may be large and frequent and consist of almost pure blood, with perhaps a few clots of a dark red, chocolate, or black colour. In these severe cases well marked symptoms usually make their appearance. The temperature may suddenly drop and the pulse become small and feeble, while the hands and feet grow cold and the face pale and anxious. The patient is often restless, and, if the loss of blood be very sudden, may have a fainting attack. It should be remembered that all these symptoms may appear while the blood is still retained in the bowel and before it is seen in the stools. In the treatment of these cases we have two objects in view, first to stop the bleeding and secondly to revive the patient and combat the weakness and anæmia that often ensues. He should be kept absolutely at rest, with the head low, and disturbed as little as possible. The food should be given iced and in small quantities; and it is well to limit the diet for a time to such articles as whey or albumen water, which are completely digested and absorbed, and are therefore not likely to excite an action of the bowels. To check the movements of the intestine and so to keep at rest the part from which the blood is coming, it is usual to give opium either by the mouth or by enema. This is done to allow the blood to clot and so block up and close the hole in the injured blood-vessel. At times the application of an ice-bag or iced compresses to the abdomen will prove useful. While the hæmorrhage is still going on, and for some time afterwards, stimulants should, as far as possible, be withheld, for they increase the force of the heart, and so tend to prevent the formation of a clot in the bleeding vessel. If, however, the pulse becomes very weak and signs of collapse appear their use may be necessary, and in such cases much immediate benefit often follows the injection under the skin of a pint or so of warm salt solution (ʒ i. of common salt dissolved in a pint of water, sterilised by boiling, and given at a temperature of 99° F.).

*Perforation* of the intestine is the most dangerous of all the complications of enteric fever and the patient, unless quickly relieved by operation, has little or no chance of recovery. Its prevention is one of the main objects of our treatment, and is the chief reason why the diet is so carefully chosen and why aperients are not allowed. For the same reason the patient should be prevented from exerting himself in any way and should always be moved carefully and gently. Especially is it important to protect the abdomen from pressure or rough handling while washing, moving, or changing the patient. Like hæmorrhage, perforation most frequently occurs in the third week or a little later. Its onset is generally acute, the first and most usual symptom being sudden, sharp pain in the abdomen. There may be a rise or fall in the temperature, and not uncommonly there is a slight shivering attack or even a well marked rigor. The pain persists more or less continuously and may be accompanied by vomiting. The abdomen is tender and soon becomes distended and the patient rapidly sinks. He lies with his legs drawn up, the pulse quick and feeble, the face sunken and anxious, and perhaps covered by a cold clammy perspiration. These later symptoms are caused by the peritonitis that results from the entrance of some of the contents of the intestine into the peritoneal cavity. Frequently the onset of perforation is much less acute and the symptoms may be very indefinite, especially if the patient is delirious or unconscious. There may be only trifling pain and little or no vomiting or abdominal tenderness.

The only treatment that affords any chance of recovery is the performance of an operation, by which the abdomen is opened and the hole in the intestine exposed and sewn up. Success will depend very largely on doing this as early as possible, for the inflammation spreads very rapidly over the peritoneum, and the patient as rapidly weakens. It is therefore necessary to be constantly on the watch for any change in the patient's condition, sudden or otherwise, so that the earliest signs of perforation may be detected, and no time lost in preparing for the operation. The nurse should note and report at once the occurrence of any of the following symptoms:—abdominal pain, vomiting, hiccough, shivering, sudden rise in pulse or respiration, unusual sweating, or any signs of collapse.

*Urinary Symptoms.*—During the early stage of the disease retention of urine is not uncommon, *i.e.* the patient is unable to pass urine, and it may be necessary to relieve the distended bladder by means of the catheter. It will be required two or perhaps three times in the twenty-four hours. Great care must be taken to keep the catheter scrupulously clean. It should be well washed out with water after use and then sterilised by boiling. The nurse should always note whether urine has been passed or not and report accordingly. As a rule the patient regains power over his bladder in a few days.

During the height of a severe attack there is frequently more or less incontinence of urine, *i.e.* the urine is passed involuntarily and perhaps without the patient's knowledge. There is no way of preventing this. The nurse should report it, and at the same time redouble her precautions against bed-sores, never omitting to remove the clothing as soon as it is soiled.

Inflammation of the bladder sometimes occurs and may result from the use of dirty catheters. The urine then becomes thick and foul smelling, and may contain pus and give an alkaline reaction. In such cases it is sometimes necessary to wash out the bladder.

Throughout the disease the urine should be frequently tested. It often contains a little albumen. Occasionally acute nephritis occurs as a complication, and the urine is then of a red or smoky appearance, due to the presence of blood.

*Mental Symptoms.*—More or less mental disturbance is common. The patient is usually dull and apathetic, sleeps badly, and is frequently delirious. In ordinary cases the delirium is comparatively slight and of a quiet type, and the patient can be roused sufficiently to give fairly intelligent answers to questions. In other cases it is noisy and even violent and difficult to restrain, and in heavy drinkers may take the form of delirium tremens. As a rule, in severe cases, when there is great exhaustion, it is of the low muttering type, the patient being almost unconscious, but restless and constantly muttering to himself. Usually it is most marked when the temperature is highest, and any treatment that reduces the temperature tends at the same time to remove the mental disturbance. If there be much restlessness the undivided attention of one nurse may be required, and it is a good plan to fix padded boards to the sides of the bed so that the patient may be more easily prevented from getting out.

In some cases, especially if the temperature is not much raised, the patient may be clear-headed throughout. This is not altogether an unmixed blessing, for he is then able to realise his helplessness and perhaps his danger, and the monotonous character of his illness may become very wearisome. Even if he has no anxiety about himself he may be constantly worrying about his family, and dreading what may happen to them if he does not get well. This occurs more particularly with young married men and is a very serious obstacle to recovery. Much will depend on the nurse. She should be cheerful and sympathetic, always looking on the bright side of things, and taking it for granted that ultimate recovery is certain. Her influence may be able to soothe the patient and relieve his anxiety when drugs or anything else would be harmful.

### The Nurses' Clinic.

### INTUSSUSCEPTION. BY A MATRON.

There are several important points to remember in nursing cases of intussusception in infants. When the baby is admitted, be careful to ascertain from the mother the exact times: when the baby began to cry out, as if in pain, when its bowels were open last, when it began to be sick, the charactor of the vomit, at what period it changed from milky to yellowish green, if she noticed any offensive smell about the sickness, and if this seemed to her like vomited stool. When did the baby begin to pass blood and mucous per rectum, and how often? Ask her to leave for the doctor's inspection one of the blood-stained diapers and clothes containing marks of the sickness. What treatment has the baby had since the onset of the symptoms? Also ascertain from her what she feeds the baby on, and if that food agrees with it. This is important for the after treatment of the patient, for it is best not to make sudden changes in a baby's diet when it has undergone such a serious operation as the one it has to face.

Before the operation no food of any kind must be given. The child will, as a rule, be very collapsed after admission, so—anyhow in cold weather—it is well to put the bed in front of the fire and give plenty of hot bottles and light blankets. Heavy bed clothes, like quilts, must be placed so as not to press on the baby's abdomen; this is done by means of a cradle, or, as this often fidgets a baby, by laying the bed clothes over the rails of the bed. If allowed, the best way to revive the baby is to give it a nice hot bath, and then dress it in suitable clothes. A very loosely knitted vest and a long flannel gown, open at the back, are most suitable, because they are easily taken off and put on again.

If the operation has been decided upon, the nurse can now—with the doctor's consent—purify the little patient in the usual way, being careful to sterilise a large area up to the waist and half way down the thighs on both sides. This is important, because a surgeon, used to the large surfaces of adults, will easily expose half the baby. Special care must be taken to remove all dirt from the folds of the umbilicus, and this is best done with a rough probe, covered with wool, soaked in methylated ether. The nurse must also remember that she is handling very delicate skin, and not scrub harshly, but rather gently massage in with her own hands the ether soap for a good while. The compress of lotio. hyd. perchlor. 1 in 2,000 should be applied as hot as a fomentation, and will thus act, not only as a disinfecting agent, but also as a means of allaying pain. Immediately before the operation the nurse must pass the catheter and test the urine. No enema or other means of trying to induce the bowel to act must be attempted by the nurse. The baby's legs and arms are wrapt up in cotton wool, a little space being left on one wrist to expose the radial pulse. Before taking the patient to the theatre the nurse prepares the bed for the baby after the operation. For this purpose a second bed is often available, or the baby can be laid on another bed for the time being. The bed for a case of intussusception is made up with a divided mattress, a bedpan covered with a ringwater cushion being inserted in the hollow space, placing the handles downwards and outwards so as not to interfere with the baby's legs. The rim of the water-pillow is covered with a hollowed-out small sheet. The top bedclothes are folded back ready on the top of the rails, the sides being let down. Four hot bottles are placed on the part of the bed where the baby is going to lie, the bed rolled in front of the fire, the foot raised on high blocks, and three blankets and a head flannel put before the fire. On the bedside locker, well-fitting chest straps, pieces of bandage, and a towel should be ready. The nurse must also prepare a feed in a bottle and take it to the theatre with the patient. This feed consists generally of about one ounce of very diluted ordinary or peptonised milk, and must be kept warm in the theatre. Here the nurse takes off the baby's clothes as for other operations on the trunk, and covers it up with warm blankets when it has been put on the well-warmed table. Before the sterile towels are placed over the patient, she should ask the "clean" nurse to put a piece of sterile wool over the baby's chest. Otherwise the nurse has her ordinary duties of assisting the anæsthetist to perform.

At the close of the operation, when the little patient is often very collapsed, we have found that nothing will revive it as quickly as the teat of a feeding-bottle, placed between its lips. If there is any life left in the baby, it will respond and suck, and the mere act of sucking acts as an excellent stimulus and counteracts the collapse very effectually.

On being put to bed, the infant is immobilised with straps across the chest and to the legs. If it is at all inclined to be fretful we give it a boiled comforter—for it is better to tolerate this eyesore than to have to resort to strong drugs to induce a baby to keep quiet during this critical period. Feeding, as a rule, presents few difficulties. We generally begin to feed the baby immediately after operation with a smaller quantity than is usually given for its age, but we never starve cases of baby intussusception, like ordinary abdominal cases. A baby who has already, through disease, had no food for 24 hours or more, would ill stand such a prolonged curtailment. Unless we let the baby have its accustomed home mixture, we feed it on peptonised milk or Benger's food for two or three days and then go on to the ordinary milk mixture.

The baby must be kept extremely quiet, and disturbed only when absolutely necessary. The bed is put level as soon as the shock has subsided. The condition of the bowels must be carefully watched and reported; a little blood and mucous during the first 24 hours need not alarm the nurse, as long as defecation is otherwise re-established, neither need a rise of temperature, even to 103, cause anxiety during the first 24 hours. Babies have a way of responding rather more forcibly to interference than their elder brothers.

### American Nursing Notes.

### MONEYED PIONEERS.

Nursing in the United States is threatened with a danger which the more enlightened spirits are beginning to recognise. Amongst them are a good many ladies who are said to possess considerable private means, and are, therefore, quite able to maintain themselves in comfort, if not in luxury, without working at all. Some of these ladies have thrown themselves into the nursing movement with great zeal, but they seem not to have realised the conditions which prevail in the case of those nurses who have little or no private means, and are dependent upon their earnings for their maintenance. Naturally wealthy pioneers are often very ambitious. One idea of theirs is, that each of the great universities should establish a college of nursing; that is a separate department, like that of law, medicine or arts, with a separate curriculum, and a four years' course to include hospital experience and private nursing. They claim for nursing, that it shall be recognised as a learned profession, and this aspect is put forward energetically as the highest ideal to be attained. Meanwhile, claims are advanced, that the training of nurses, and all that appertains to its efficiency and success, should be undertaken by women alone, who should be left entirely free to work out their own ideals independently. That is, as we are advised, independently of the governing authorities of the institutions which provide the money and the means, whereby nurses secure hospital training. We can well understand that the medical profession in the United States and the governing authorities of some of the most important hospitals are beginning to recognise, that unless some limit be placed to the ambitions of the more enterprising leaders of the nursing movement in the United States, troubles must arise, which may lead to a reconstruction of the conditions, which prevail at the present time, in regard to nurse-training schools.

### Miss Dock as a Leader.

Miss Lavinia L. Dock, who is one of the ablest and most experienced of the wealthier pioneers of the nursing world in the United States, has spent a good deal of time in Europe studying nursing methods, and is believed to be largely responsible for the advocacy of these advanced nursing ideals. Of course all reformers must cast their net as widely as possible at the outset in order to secure continuous progress. But Miss Dock will, we believe, be the first to recognise, that success can only be hoped for, when the greatest discretion in advocacy is exhibited by those immediately responsible for the policy of reform. We should, therefore, welcome a statement from Miss Dock defining exactly the position and aims, which she and her friends are working, so zealously, to secure and promulgate.

### Nursing Homes, Nurses and Practitioners.

Several years ago in this country, the conductors of one of the larger nursing homes recognised, that regulations were desirable, to protect the interests of the private medical practitioner who sent cases there under the care of a consultant. Patients who became attached to their nurses, on attaining convalescence, were often anxious to take their nurse with them to the seaside, or wherever they might be going on leaving the nursing home. In practice this new habit tended to divorce the patients from the private medical attendant; and there was danger lest he should be supplanted in the good opinion of the former by the consultant and the nurse. In the United States there is evidence of a similar danger. We take the following story from the current number of the *American Journal of Nursing*:—"A family physician being unable to send his patient an 'experienced nurse,' apologised for substituting a trained nurse. In the result, when the patient again became ill and the doctor promised to send an experienced nurse, the patient said, 'Oh no, doctor, no more experienced nurses for me! I prefer them trained.' When, in surprise, he demanded to know the reason why, she replied, 'Because I noticed that when I had an experienced nurse you came always once a day, sometimes twice, but when I had a trained nurse you only came every day during the very acute stage of my illness; then two or three times a week was sufficient. I paid the trained nurse a little more, but I paid you a great deal less. I had infinitely better care, and the total cost of my sickness was much less than it would have been, with an experienced nurse, and daily or twice daily visits from you.' "

The Registration Deadlock in New York.

Nurses working in New York State are beginning to realise at its proper value, the dangers arising to themselves from the hurried rushing through the State Legislature of an Act for the Registration of Nurses. We have always contended, apart altogether from the merits of the question, that no legislation on this subject could possibly prove satisfactory which was hurried and ill-considered. The New York experience proves this contention to the hilt. When registration was first mooted in New York and a Bill was drafted, it was publicly stated, over and over again by the promoters, that " all graduate nurses of good standing, who could give evidence of a two years' training, would be eligible for registration without examination." When, however, the Registration Act was passed, it was found, that it differed materially in many respects from the Bill, and that it, in fact, did not fulfil the promises of the original promoters. In the result, a large body of nurses of good standing, who can give evidence of two years' training and general efficiency, are being refused registration, on the plea, that the schools, where they were trained, as things exist to-day, under a Registration Act, are not now up to the standard.

In England, matters would probably prove infinitely more unjust to a large body of working nurses of good standing, seeing that by far the majority of the nurses on whom the profession and the public have at present to depend for nursing the sick, might practically be shut out from registration. That is why it is essential that no Registration Act shall be passed by Parliament for some years to come at any rate. In New York, the State Nurses' Association is taking steps to amend the Act, for, unless they succeed in doing so promptly, the whole registration movement there may be set back, if it is not, in fact, stayed altogether. How critical the situation is may be realised from the circumstance, that only 900 applications for State registration have been made in New York during eighteen months, and the Act, as it stands, is reported to have caused many hundreds of nurses to organise against the whole movement of registration. We shall watch developments with great interest. Meanwhile English nurses and all interested in their welfare, including members of both Houses of Parliament, may properly decide, not to allow any Registration Bill for nurses to be passed, until matters are so organised, as to guarantee, in advance, justice to every nurse of good standing and competency.

A Government Inspector of Training Schools.

Our American cousins are proverbially noted for not allowing the grass to grow under their feet. It is only quite recently that Maryland legalised State registration for nurses, but the Governor has promptly appointed Miss G. C. Ross of the Johns Hopkins Hospital as an inspector of training schools. We understand, that without loss of time, Miss Ross has made her first inspection of all the training schools in the State of Maryland. The incident illustrates one of the consequences which is likely to follow the enactment of State registration, in these islands, by Act of Parliament.

### Nursing at Ober Ammergau Cottage Hospital.

Nature has done much for Ober Ammergau. The air is invigorating; visitors wonder that anyone can ever be ill; but alas! there are the usual sufferings in this favoured valley, and especially ailments that need clever treatment and good nursing. The natives are talented in many ways, and while the men devote themselves to wood carving, the women do a great deal of the field work, for here there is peasant proprietorship, bringing with it hard labour and, too often, scant returns. The women grow old before their time, and the young girls are subject to a serious ailment arising from carrying heavy weights on the hips.

The Origin of the Hospital.

It is not generally known that after the heavy expenses of the decennial recurrence of the Passion Play are paid, the Government of Bavaria demands that a large proportion of the surplus be spent on some public work for the good of the village and neighbourhood. In 1890 part was to have been spent on a new organ for the church, but the English people, through the instrumentality of the late Mr. Evans, who devoted himself to achieving this object, presented Ober Ammergau with a beautiful organ. Then the Government suggested that barracks should be built, but the Ober Ammergau people pointed out that this would be a very undesirable addition to their peaceful village, and suggested a cottage hospital, which was accordingly erected for the benefit of the community and the immediate neighbourhood. It is a plain building, very unlike the picturesque cottages that cluster round it, but roomy and comfortable.

The Patients.

There are beds for 21 patients, and a sister of the Red Cross attends to the sufferers. She has very little assistance, but, visit the hospital when you will, everything is in order. The patients look as happy as their sad circumstances permit. Everything is clean and sweet. In fine weather those who are well enough lie on couches in the glorious sunshine, drinking in the pure air from the neighbouring mountains, the sister visiting them and bringing them their meals and medicine. The system in Germany which compels employers of labour to put by a certain sum weekly in case of accident or sickness—a similar obligation is laid on all employés, including domestic servants—secures those in need a free pass into the hospital. Other patients have to pay 3 marks (English shillings) a day. There are many cases that could be nursed at home, but it will be easily understood that the sister in charge of the hospital could only visit a case of life or death, and not always that, when her hands are more than full in the hospital. The people themselves are wonderfully helpless in case of ordinary illness. A few friends of Ober Ammergau during the Passion Play of 1900 presented an operating table and some valuable cases of instruments to the hospital.

A Second Sister Needed.

A larger scheme occurred to a few devoted friends, who felt that some substantial benefit to the whole community could only adequately express the debt of gratitude the world owed to the actors in the great drama, which sets forth so vividly the story of the Saviour's sufferings and His great sacrifice for the sins of mankind. These friends started a subscription in order to endow the hospital of Ober Ammergau with a second sister, who would live in the hospital and assist the present sister, but whose daily work would be to minister to the sick in their own homes. Then the Burgermaster, the beloved and honoured Josef Meyr, gratefully accepted the proposal in the name of the burghers, and for five years the work of collecting the necessary sum has proceeded. The idea was that everyone who had been at Ober Ammergau since 1870, 1880, 1890, and 1900 should have a chance of helping. His Majesty King Edward sent a generous donation with a kindly word of commendation of the scheme. The Duke of Connaught also subscribed, and it is hoped that the required sum will now soon be completed. It is not a charity in the ordinary sense of that too-often misapplied word. It is a free gift from England, and it should not rob a single home charity of a single shilling. During this summer a great play will be given at Ober Ammergau called "The School of the Cross," and the earnest hope is entertained that the many visitors will contribute to the fund for providing a nursing sister for Ober Ammergau in memory of the Passion Play and of Josef Meyr the great exponent of the principal part in the drama filled by him in 1870-71, 1880, 1890, and 1900. He entered into his rest on St. Andrew's Day, 1903, after a painful illness borne with Christian fortitude, and no better memorial could be raised to a noble life.

### Select Committee on the Registration of Nurses.

EVIDENCE OF MISS LÜCKES.

On Thursday last week Miss Lückes, matron of the London Hospital, was the only witness examined before the Select Committee of the House of Commons.

The Chairman said that the committee did not want to travel over the rather well-worn ground covered by former witnesses; they would, however, be glad to have Miss Lückes' views on several points. They recognised the claim of the London Hospital to a very efficient training, but they would like to know why Miss Lückes in her précis deprecated uniformity? He suggested that the best way of eliminating the undesirable nurse (whom everyone agreed did exist), and meeting the demand, which was greater than the supply, was by turning out as large a number as possible of nurses, able to take any case to which they might be sent. Three years seemed to him a long enough period in which to render them efficient in this sense. It was a very unfortunate circumstance that hospitals sent out nurses who were not trained for every case. He wished all the training schools were as good as the London; there would be fewer bad nurses. Did Miss Lückes not think that a "hall-mark" would tend to increase the supply of efficient nurses? And what was there to prevent the State giving a similar certificate to that given by the London Hospital or by the matron of any good training school? There need be no suggestion of uniformity; the certificate, like that of the London, which is graduated as to degrees of efficiency, would mean neither more nor less than what was stated on it.

Miss Lückes said that she deprecated uniformity because nurses were wanted for every variety of case and circumstance. It would be an immense waste to put every nurse through not only a general but also a special training. Nurses were very well trained in country hospitals for the work they had to do, and that training fitted them far better than such a comprehensive scheme as had been suggested by some advocates of uniformity. There was room in nursing for many varieties of individual, and this, she held, would tend to be lost sight of if uniformity in training—a great mistake, in her opinion—were insisted upon. The best way to eliminate the undesirable—the number of whom she believed to be very greatly exaggerated—was by producing as many as possible of a better type. Complaints of "unsatisfactory nurses" frequently proved in reality to refer to the same nurse over and over again, her reputation being handed on from one to another; and, though not wishing to minimise the gravity of the situation, she desired to point out the tendency for one case to be multiplied. The great evil was not ignorance, and the subject of complaint was not nearly so often incompetency as personality; the "upsetting of the patient," selfishness, or objectionable manners being the most common causes. In order to produce enough nurses of a better type, it was not necessary to insist on a three years' training: this had been elevated by many people into a fetish of late years, and she held very strongly that while a minimum of training was an undoubted necessity, a woman might be quite fit to take a responsible position in a very much shorter time than three years. It was an injustice to make such a woman "fill up time" merely to comply with a "three years' course." Character was of far more importance in a nurse than technical qualifications; anybody could gain a certificate, but everybody could not be a good nurse. A woman might be of a kindly disposition, and yet unable to distinguish herself in theoretical or in special (*e.g.* surgical) nursing; such a woman should be sent to chronic cases, which chiefly required kindly attentions, while a smart clever woman, although without those gentle qualities, might be very suited for a surgical case requiring above all technical skill. Of this the hospital, not the State, was the best judge. For this reason it would be a fatal mistake to "hall mark" every nurse, regardless of the kind of case which she would be likely to attend. But the real difficulty was lack of accommodation; the hospitals could not turn out a sufficient number of trained women until this was remedied. The result was to make the work of the nurses much harder, and to give them less off-duty time than they ought to have. She would proceed by providing increased accommodation so as to train in larger numbers.

The choice of candidates, Miss Lückes said, must not be judged from the number of advertisements appearing in the papers; these frequently referred to the same individual. Nurses of the right kind were scarce, and to insist on a three years' course would still further cut short the sources of supply. A State certificate would not increase it, and it would tend to stop inquiry. At the London Hospital a register was kept as far as possible up to date, but inquiry was advised in doubtful cases. It had been said that it would be possible to keep a record of a woman's career, and to certify that she was "living a life fit for her work." She could hardly imagine what that would mean—in the number of inspectors, knowledge, judgment, salaries—to do this. Among the public there was a growing and hopeful tendency to inquire of the hospital whence the nurse came, which it would be a great mistake to check.

In reply to Sir John Batty Tuke, Miss Lückes said that uniformity imposed by the State could be only nominal; the standard even of a Central Board might change. She could not see what use the public would make of a register; a directory might of course be published, but it would not ensure the securing of the best nurses; it would not even follow that the best were enrolled, although it would be to the nurse's interest to be on the list. The London Hospital practically recognised two classes of nurse, but it would be a great mistake for the State to do so; the public would be deluded into thinking every nurse recognised by the State a good one, the minimum so readily crystalised into the maximum. It was too early yet in the history of nursing to stereotype. She did not think that there were enough incompetent nurses to make drastic legislation necessary; indeed, the subject did not commend itself to her as adapted to legislation at all. The profession was in a position of very hopeful vitality, and it would be a thousand pities to arrest the development.

The sittings were then adjourned until May 11th at 11.30, when further evidence will be taken.

### A Book and its Story.

### MRS. HUMPHRY WARD'S NEW NOVEL.

"The Marriage of William Ashe"—the title of Mrs. Humphry Ward's latest novel—is a book which has provoked some diversity of opinion from a literary standpoint. As a whole, it is neither so powerful nor so brilliant a study of contemporary society as a previous one by the same author, "Lady Rose's Daughter." But it has many points in common with it. The heroine, Lady Kitty Bristol, is also of doubtful parentage; she is surrounded by a circle of notable people socially and politically, who, under their thin disguise, are easily recognisable, for they move in a period nearer to the present than the scene of Lady Rose's romance, and she is a gifted, wilful, and wholly unconventional character. Education in a French convent has been powerless to suppress an apparently irrepressible personality. She has a heart, true in its first instincts, but vanity and waywardness and absence of self-control lead her away. William Ashe, a young and rising statesman, meets her at a reception in St. James's Place, given by her mother, Madame d'Estrées, a lady whose beauty and fascination were as notorious as her lack of morals. Lady Kitty, her daughter, is supposed to be the child of Lord Blackwater, her first husband. On this particular evening, at a gathering where men predominate, Ashe is attracted by Lady Kitty at once. His introduction is followed by the announcement from a friend—"Lady Kitty, Mr. Ashe is an old friend of your mother's. Congratulate him; he has just got into Parliament." Ashe receives a characteristic reply from Lady Kitty, "I never congratulate anybody till I know them." "How long must I wait?" "That depends. Are you difficult to know?" He then asks her if she had ever known anyone confess to being easy to know. "Well, I'm easy to know," she said, carelessly, leaning back, "but then I am not worth knowing." This informal greeting between the two ends, after a few weeks' further acquaintance, with the sudden determination of William Ashe to make Lady Kitty his wife. From the first, in addition to the very real attraction of her volatile personality, the chivalry of a nature, not too easily stirred, had been aroused by the forlorn position of Lady Kitty in her mother's house. Her presence there, coupled with the knowledge of her mother's reputation, and a sense that between them, on her father's side, lay a claim of kinship, moved very deeply this man, who is described as a being of "ironic moods," with the reputation of an idler, though in truth he was an unwearied student. He realises the absurdities of his own class more plainly than the absurdities of the populace, and in addition, politically, he is an aristocrat immensely interested in liberty. A member of the Government, and proud to be an Englishman. Lady Kitty was opposed in every respect, in appearance and manners, to the conventional type of young English womanhood he had, so far, met. Early Victorian girlhood was very unlike this irresponsibly impulsive little being who appealed to him so keenly by the force of contrast. "She looked at him audaciously, and he on his side could not take his eyes from her, so singular was the small sparkling face. The hair and skin were very fair, like her mother's; the eyes dark, and full of fire; the neck most daintily white and slender, the figure undeveloped, the feet and hands extremely small. But what arrested him was, so to speak, the embodied contradiction of the personality—between the wild ntelligence of the eyes, and the extreme youth, almost childishness, of the rest. . . . Extreme youth, innocence, protest, pain: was it with these touching and pleading impressions, after all, that his first talk with Kitty Bristol had left him?" Quite soon after their first encounter Ashe goes down to join a Lady Grosville's house party in Cambridgeshire. He finds from a cousin who was in love with him—Mary Lyster—and who, up till this time, had been one of the many women rumour had set apart as his future wife, that Lady Kitty was to be at Grosville. "In the omnibus, on the way to Grosville Park, he and Mary gossiped in a corner, while the Cabinet Minister and the editor went to sleep, and the two Members of Parliament practised some courageous French on the Austrian Attaché." Ashe learns that the invitation to Lady Kitty had not been given without some misgivings on the part of Lady Grosville. "Yes, poor child, I heard from the Grosvilles, she was to be there," Mary Lyster said; "Why 'poor child?' I don't know. It should be 'poor hostess.'" "Oh! the Grosvilles complain?" "No. They're only on tenterhooks. They never know what she will do next." "How good for the Grosvilles!" "You think society is better for shocks?" "Lady Grosville can do with them any way. . . . But I'll back Lady Kitty." "I haven't seen her yet," said Mary. "I hear she is a very odd-looking little thing." "Extremely pretty," said Ashe. "Really," Mary lifted incredulous eyebrows; "now, I shall know what you admire." "Oh! my tastes are horribly Catholic—I admire so many people," said Ashe, with a glance at the well-dressed elegance beside him. Lady Kitty's escapades at Grosville during her brief visit were sufficient to verify the fears of her hostess. In spite of them she attracted all the men of the party and at the close of Ashe's stay he decides that if he is to propose to Lady Kitty no time is to be lost. Among her admirers is the dean, who was very much a man of the world, and had spent his boyhood in Paris, where his father had, under Louis Phillipe, been the British Ambassador. He falls under the spell of Lady Kitty's fascination from a graver side.

They talked together of past and present French art, literature, and events in a world to which she was no stranger. She even consented to recite Corneille. It was Sunday evening, "Her beautiful head, her kindled and transformed face, her little hand, on the white folds of the lace cloak, these alone remained to mingle their impression with the austere and moving tragedy which her lips recited. Her audience looked on at first with the embarrassed or hostile air which is the Englishman's natural protection against the great things of art; then for those who understood French the high passion and noble verse began to tell; while those who could not follow were gradually enthralled by gestures and tones, which suggested and conveyed the finest and most compelling shades of love, faith, and sacrifice." In spite of frank avowals on her part of bad temper of an ungovernable type, which would make him miserable, of overpowering fancies, which she must follow at times, of moods in which her head seemed on fire, Ashe asks her to marry him and she, after some uncertainty, accepts him. Then follows the story of his married life. A life in which Lady Kitty moves, in spite of her brains and brilliancy, as a thwarting, hopeless influence on his public career, which nothing apparently could check. His affection never wavers. He had married her with open eyes and blamed no one for the result. The book grows in force as the story advances. This is not the place to give away the key to the situation. But William Ashe's marriage is an object lesson, whether intended as such, or not, on the folly of public men choosing for a wife a woman who has not the saving gift of reticence, and the fine tact necessary to her position.

* The Marriage of William Ashe. By Mrs. Humphry Ward. Smith, Elder. 6s.

### Everybody's Opinion.

[Correspondence on all subjects is invited, but we cannot in any way be responsible for the opinions expressed by our correspondents. No communication can be entertained if the name and address of the correspondent are not given as a guarantee of good faith, but not necessarily for publication All correspondents should write on one side of the paper only.

### A NOVEL PROPOSAL.

"An American-trained Nurse" writes:—I regret that nurses, trained or untrained, should so lack refinement as to abuse each other through the columns of a widely-read paper, or indeed through any other medium. Charity covers a multitude of sins. But is it not possible that Nurse E. Woodburn Heron's remarks refer to nursery maids, who also wear our uniform, and cannot be expected to show refinement even in a nurse's garb? If hospital trained nurses would take steps to protect their uniform, people lacking refinement would not be able to throw the sins of others on our already overburdened shoulders.

"Another Trained Nurse" writes:—The argument of your correspondent signing herself "Nurse E. Woodburn Heron" is somewhat difficult to follow. (1) She writes that she has seen women in shops, very untidily dressed, wearing nurses' uniform, whom she "presumes" are trained nurses. Why? She herself writes as an "outsider"; by her own showing she is not a trained nurse, and yet "always wears uniform," so she must be well aware that its use is not confined to trained nurses. (2) She disapproves of the outdoor uniform of "three large London hospitals" as being "against the first and most simple rules of nursing." And therefore she argues that trained nurses should not complain if untrained women adopt their dress and pass as "nurses." It would be as logical to say that because there are some soldiers who are untidy and slovenly when off duty, or because some irresponsible person pronounces the uniform of their regiment to be against the first and most simple rules of soldiering, therefore no one should object to civilians wearing military uniform and posing as soldiers. Fortunately there is a law preventing "the treating of his Majesty's uniform with contempt"; would that the uniform of another fine profession could be equally safeguarded!

Nurse Amy Smith writes:—I cannot let Nurse Woodburn Heron's scathing condemnation of the appearance of trained nurses in London pass without making some protest on behalf of my fellow trained nurses. Although I did not receive my training in London, but at one of the largest provincial hospitals, I have been working in London for the last three years, and my experience has been nothing like your correspondent describes. I think, considering the hundreds of nurses belonging to the different London hospitals and institutions, you will find very few in the almost degrading state that Nurse Heron describes; she must have seen, unluckily the "few swallows that do not make a summer." I know several Bart.'s nurses, as I do nurses belonging to other hospitals, and should in no way exalt their appearance above that of many other trained nurses; their "bows" may be smaller, but I do not think that their linen is any more spotless, or their general appearance more professional. Nurse Heron owns to not being a trained nurse. Does she think that the nurses she has described would be allowed in the wards of a hospital? One may get splashed and dirtied on a wet day; but dirty dresses and aprons would have to be changed before going "on duty," and I do not think any matron or sister would tolerate nurses who had untidy and dirty hair. A private nurse may have a heavy case, and after being on duty all night may be only able to take an hour for out-door exercise. Then she is not able to spend the short valuable time in making her appearance all she would wish, with the risk of losing her one chance of fresh air; but even here there is no excuse for dirtiness. I sincerely hope that other trained nurses in London will raise their protest with mine against the unjust sweeping assertions of Nurse Woodburn Heron.

"A Jay-Pea" writes:—May I make another suggestion in connection with the grievance alluded to by "A Trained Nurse" in your issue of April 8th? In our Navy all the officers wear the dark-blue cloth, gold lace, and buttons by which they are recognised all over the world, beginning with the middy with the buttons only on his cuff, rising, through the sub-lieutenant with his single band of gold lace to the admiral with his four. Could not some such expedient be adopted in the nursing profession, beginning with the cottage nurse who has merely been trained in simple maternity work, rising through those who have taken their midwife certificate after the minimum of training required for it, to the fully-certificated nurse in all branches of her profession with four, five, six years' training if she likes adding a band for each year? If this were a recognised plan and universally adopted on the cuff, many difficulties would be got rid of, and jealousies would vanish, because each grade would have her proper place in her profession. The middy does not expect to be given a captain's command, and would know he could not fulfil its duties except in a case of emergency. So the simple maternity nurse would not be called upon, as she often is in country districts, to take charge of complicated cases and operations, or if she is, for lack of a more suitable person, it would be with the distinct understanding that it was because no one else was available. Her cuff would protect her from invidious comparison with her more highly-trained sisters. One difficulty I experience in managing a country nursing association of simple cottage nurses is that doctors, having no other nurses at hand, frequently use mine for operations, etc., for which their training was never intended to fit them. I should be very glad of some simple expedient which would clearly distinguish them from the highly-trained nurse. In all the papers I have read and speeches I have heard, it is always taken for granted that the short-trained nurses are trying to pass themselves off as equal to the first-class nurse, which is quite a mistake. We do not for one moment think ourselves equal to a three years' trained nurse, on whom, by the way, the four years' trained person is beginning to look with contempt. With regard to those who have had no training at all, and I know a case in point, the nurse has had years of experience, and wears cloak and bonnet. Let them retain these, but forbid them the "buttons" and "gold lace," just as the petty officers are distinguished at once by any one who knows the least about it. How my plan is to be carried out I do not pretend to say. I do not see that the selling of medals at one shop would be much use. What certificate would be recognised as qualifying for a medal, and who is to decide that knotty point? A universal and loyal consent among the nurses themselves is an ideal idea, but I fear that it is Utopian. We do not all insist on being doctored by a first-class London physician; most of us cannot afford it; and, in the same way, let the humbler, less costly nurse have her place, just as the country practitioner has his. He knows his limitations if he is worth anything and she knows hers.

### Presentations.

West Suffolk Hospital, Bury St. Edmunds.—Miss Lilian Heather, who has resigned her post of sister of female wards and theatre at the West Suffolk Hospital, was the recipient on Wednesday last week of a testimonial, consisting of a complete toilet set of solid silver. The gift was subscribed to by the medical staff, matron, nurses, and servants.

### Death in Our Ranks.

We regret to hear of the death of Miss Florence Marion Burnett, sister at the Hospital for Consumption, Brompton which occurred last week, from pneumonia and pleurisy. Miss Burnett was thirty-six.

### Appointments.

[No charge is made for announcements under this head, and we are always glad to receive, and publish, appointments. The information, to insure accuracy, should be sent from the nurses themselves, and we cannot undertake to correct official announcements which may happen to be inaccurate. It is essential that in all cases the school of training should be given.]

Alton Workhouse Infirmary.—Miss Martha A. Whitehead has been appointed superintendent-nurse. She was trained at Ashton-under-Lyne Union Hospital, where she was afterwards charge-nurse.

Chelsea Hospital for Women.—Miss Lilian Heather has been appointed charge nurse. She was trained at the West Suffolk Hospital, Bury St. Edmunds, and has since been night sister, sister of female wards, and theatre sister in the same institution.

Hertford British Hospital, Paris.—Miss S. Davidson has been appointed matron. She was trained at the South Staffordshire Infirmary and Eye Hospital. She has since been charge nurse at the Fever Hospital, Hither Green, London; superintendent nurse at the Union Infirmary, Newcastle-under-Lyne; night sister at the Derbyshire Royal Infirmary, Derby; and matron of the Whitworth Hospital, Darley Dale, Matlock.

Kilmuse Convalescent Home.—Miss Mary Brown has been appointed matron. She was trained at the Western Infirmary, Glasgow. She has since been charge nurse and night superintendent, *pro tem.*, at the Fountain Hospital, Tooting, London; house-matron at the Downs School, Sutton; and matron of the MacKelvie Isolation Hospital, Oban.

Margate Cottage Hospital.—Miss Gertrude Webster has been appointed nurse-matron. She was trained at St. Marylebone Infirmary. She has since done district nursing at Bolton, and has been ward and theatre sister at St. Pancras Infirmary South London.

Roxburgh District Asylum, Melrose.—Miss Helena McKinley has been appointed assistant-matron. She was trained at Guy's Hospital, London, and has since been temporary-sister at the Borough Hospital, Plymouth, night-sister at the Throat Hospital, Golden Square, London, and matron of Bexley Cottage Hospital and Dispensary. She has also done private nursing.

Royal Ear Hospital, Soho, London.—Miss Incledon has been appointed sister. She was trained at Croydon General Hospital, and has since been sister at Grantham Cottage Hospital, and junior assistant to the lady superintendent of the Nurses' Co-operation, London.

West Suffolk Hospital, Bury St. Edmunds.—Miss P. L. Lawrence has been appointed night sister. She was trained at the Sheffield Royal Infirmary, and has since been sister at Chalmers Hospital, Edinburgh, and at the Princess Christian Hospital, Weymouth.

Winchcomb Isolation Hospital.—Miss Gow has been appointed nurse-matron. She was trained at Hackney Infirmary, and has since been nurse at Hertford Hospital, Alcester Sanatorium, and Penzance Infirmary.

Workhouse Infirmary, St. Neots, Hunts.—Miss Alice E. Bond has been appointed charge-nurse, She was trained at Tendring Workhouse Infirmary.

### Wants and Workers.

Nurse Smith would be most thankful should any lady no longer having use for a large mackintosh, and a propelling chair if she might have them for a heart case, very poor, old and deserving, Longton Sick Nursing Association.

### TRAVEL NOTES AND QUERIES.

By our Travel Correspondent.

New Rockland and Quebec (District Nurse).—I fear your projected trip will cost far more than you think, though I may be wrong. Send a stamped and addressed envelope to Messrs. Cook and Sons, Ludgate Circus, E.C., and ask them the cheapest route and the exact cost of it. You must then allow a wide margin as to your purse, because living is expensive in America and tips run high.

A Month in Brittany (Summer Holiday).—August will not be hotter in Brittany than in England. The sum you have at your disposal will do the trip, but it will require management to make it last for four weeks, as you are not experienced travellers. Take second class return tickets to St. Malo, £2 1s. 2d. each. The boats start three times a week only. They arrive in the early morning. Have breakfast, and then start for Le Mont St. Michel via Pontoison. Put up on the Mount at Hôtel Poulardainé. Stay one night, or two if you like. From there go west to Lamballe. Put up at Hôtel du Commerce. Always ask for rooms on the third or fourth floor. There is not very much to be seen in Lamballe, but you must sleep there a night, trains being few and far between and very slow. Next day by an early train go on to Morlaix, a most fascinating town. Put up at Hôtel Bozellec. I think you might with advantage stay a week, there is much to be seen. Roscoff and St. Pol de Léon can be seen in a day's excursion. From Morlaix by an early train go to Lannion via Plouaret. Put up at Hôtel de France. There is much to be seen of the coast here in a single day's excursion. A diligence goes to Perros-guirec and another to Tregastel. On leaving Lannion go by diligence or private carriage to Toéguier. Stay at Grand Hôtel de France. From there by diligence to Paimpol; if you like to stay there a night or two to see more of the coast put up at Hôtel Michel. From there take train to Quingamp, stay a night at Hôtel Perissé and make an early start in the morning for Dinan, a very interesting town, where you will probably like to stay a week. Go to Hôtel de la Poste. From Dinan you can make many excursions, especially to Dinard.

Channel Islands or Rheims (C.B.).—I do not like the idea of the Channel Islands and Normandy combined. So many different places and separate resting places would come very expensive and you could not see so much properly. The interesting corner of Normandy round Cacu, Bayeux and Lisieux is enough for a fortnight without anything else. If you have a fancy for the other trip it can be done for £10 each quite easily. Second class return ticket £3 12s. 2d. each. Fourteen days at hotels at eight francs per day will be under £5, and what remains will pay your tips and excursions. At Coucy-le-chateau, there are two small inns, Hôtel des Ruines and the Pomme d'Or. At Laon go to Hôtel de la Bannières in the Rue David. At Rheims go to Hôtel de l'Europe, 29 Rue Buirette. Always ask for rooms on the third or fourth floor. Take your early breakfast of coffee and rolls and your late dinner in the different hotels, but get a light luncheon elsewhere. It comes cheaper. Tell me if you want more help.

Notice.—Remaining answers are held over until next week. Space being limited.

Rules in Regard to Correspondence for this Section.—All questioners must use a pseudonym for publication, but the communication must also bear the writer's own name and address as well, which will be regarded as confidential. All such communications to be addressed "Travel Correspondent, 28 Southampton Street, Strand." No charge will be made for inserting and answering questions in the inquiry column, and all will be answered in rotation as space permits. If an answer by letter is required, a stamped and addressed envelope must be enclosed, together with 2s. 6d., which fee will be devoted to the objects of "The Hospital" Convalescent Fund. Ten days must be allowed before an answer can be published.

### Notes and Queries.

**REGULATIONS.**

**The Editor is always willing to answer in this column, without any fee, all reasonable questions, as soon as possible.**

**But the following rules must be carefully observed.**

1. **Every communication must be accompanied by the name and address of the writer.**
2. **The question must always bear upon nursing, directly or indirectly.**

**If an answer is required by letter a fee of half-a-crown must be enclosed with the note containing the inquiry.**

*Home for Baby.*

(26) Is there a reliable home where a baby can be received from the first month?—*Nurse B.*

There are no such homes for children unless they are orphans. You had better advertise.

*Ladysmith Medal.*

(27) If Sister L. L. Shappell will send us her present address, we shall be glad to communicate with her with regard to the above.

*Free Sanatoria.*

(28) Will you kindly let me know if there are any institutions in this country for the free treatment of consumptives?—*Nurse E. E. D.*

Many institutions have some free beds. See the list of sanatoria published by the Society for the Prevention of Tuberculosis, price 7d., post free, from the Scientific Press, 29 Southampton Street, Strand.

*Home for Incurable Children.*

(29) There are, I know, homes for incurable adults. Are there any for children near London?—*Q.*

Yes, there is the Hospital and Home for Incurable Children at Northcourt, College Villas Road, Hampstead, N.W. This hospital used to be at Maida Vale.

*Training School.*

(30) Will you kindly tell me if Stockton-on-Tees Hospital is a registered training school for nurses?—*M. C.*

Training schools are not registered. At Stockton-on-Tees Hospital nurses are only trained for two years and given certificates.

Are the Norfolk and Norwich and the Brighton Hospitals good training schools? If so, what do you advise me to do? I have been trained for 1½ years, but want to get my three years' training. I am 26 years old.—*Anxious.*

Both hospitals you mention are good training schools where you can get your three years' certificate. If neither has a vacancy, write to the matrons of the principal hospitals, and you will probably find perseverance rewarded.

*Paying Probationer.*

(31) 1. What advantage has a probationer paying a premium over one who does not? 2. Is the certificate obtained by the latter as good as the former? 3. Is the training at some hospitals considered superior to others and in what way? 4. What is the maximum age for probationers? 5. Does three years general training qualify a nurse to take infectious cases, but not maternity?—*Taffy.*

1. None, except that paying probationers are often received older and younger than ordinary probationers, and for a shorter period. 2. Quite as good. 3. The training at a hospital with a great number of beds is better than that obtained where there are fewer beds, as more experience is gained. 4. Usually 35. 5. Neither fever nor maternity.

*Home for Spinal Patient.*

(32) Can you tell me where a spinal patient (trained nurse) could be received free or for small cost per week? Some open-air treatment desirable.—*M. A. H.*

There is a free convalescent home at Bognor connected with Merchant Taylors Company—address Edward Nash, Esq., 30 Threadneedle Street, E.C.—and another, Thomas Banting's Memorial Home, Parade Lodge, Marine Parade, Worthing, where she might be received, or the Sisters of St. Peter's Community, St. Peter's, Kilburn, would tell you particulars of their homes if you sent addressed envelope.

**Handbooks for Nurses.**

| | Post Free. |
|---|---|
| "A Handbook for Nurses." (Dr. J. K. Watson.) ... | 5s. 4d. |
| "The Nurses' Dictionary of Medical Terms." ... ... | 2s. 0d. |
| "Massage" (Swedish.) ... ... ... ... ... ... | 2s. 6d. |
| "Surgical Ward Work and Nursing." ... ... ... | 3s. 10d. |
| "A Complete Handbook of Midwifery." (Watson.) ... | 6s. 4d. |

Of all booksellers or of The Scientific Press, Limited, 28 & 29 Southampton Street, Strand, London, W.C.

### For Reading to the Sick.

EASTER-TIDE.

That Easter-tide with joy was bright,
The sun shone out with fairer light,
When, to their longing eyes restored,
Th' Apostles saw their risen Lord.

He bade them see His Hands, His Side,
Where yet the glorious Wounds abide;
The tokens true which made it plain
Their Lord indeed was risen again.

Jesu, the King of Gentleness,
Do Thou Thyself our hearts possess,
That we may give Thee all our days
The tribute of our grateful praise.

O Lord of all, with us abide
In this our joyful Easter-tide;
From every weapon death can wield
Thine own redeem'd for ever shield.

*From the Latin.*

He who has conquered death in His own person, will supply whatever may be our personal need at the moment of death. . . . .

The soul has many needs in dying, but it is the grace of our Lord Jesus Christ which supplies them all. Forgotten foes, old sins that have long been lain aside, fresh forms of evil that mock us, coming up for the first time, pains of the body, cravings of the soul, memories, present consciouness, hopes and fears, all have their needs; but Jesus has conquered death in his own person, when it assailed Him with all the accumulated violence that it could gather against mankind, and He who conquered lives in us to conquer still.—*R. M. Benson.*

Our separate lives are but fragments of some larger life, and that life again is Christ's. He quickens us not as individual units, but as parts of Himself. He raises us up not to stand alone, but as members of His glorified Body. He trains us while we are still kept apart from one another by the conditions of mortality to reach forward to this loftier fellowship. He communicates to us His flesh, His humanity, in which is the fulness of union; He warns us that selfishness, isolation, is death.—*F. W. Hicks.*

A dying body is adapted to the world of sense and time, a deathless spirit is meant and made for a world immortal as itself. Created eternal, it is intended, from the instant of its birth, to breathe the air of eternity. It is at home only in its own high sphere of being; connected by a visible frame with the present world, it is itself invisible, and lives by the Invisible. Through its own proper organs—through Faith, and Hope, and Love divine—it already commerces with that eternal scene, and the God of that eternal scene, where hereafter, disburdened of its earthly fetters, it is to dwell and to rejoice for everlasting.—*Archer Butler.*

Be with us in weal and woe
As we on our journey go;
Be with us in every age
Of our earthly pilgrimage;
And in death's dark eventide
May we see Thee at our side;
And when we arise, may we
Live for ever, Lord, with Thee!

*Bishop C. Wordsworth.*

